# Neurostructural Correlates of Polygenic Risk for Coronary Artery Disease in Relation to Youth Bipolar Disorder

**DOI:** 10.1111/bdi.70065

**Published:** 2025-09-24

**Authors:** Nidhi P. Kulkarni, Clement C. Zai, Kody G. Kennedy, Megan Mio, L. Trevor Young, Bradley J. MacIntosh, Benjamin I. Goldstein

**Affiliations:** ^1^ Centre for Youth Bipolar Disorder Centre for Addiction and Mental Health Toronto Ontario Canada; ^2^ Department of Pharmacology & Toxicology University of Toronto Toronto Ontario Canada; ^3^ Centre for Addiction and Mental Health Toronto Ontario Canada; ^4^ Department of Psychiatry University of Toronto Toronto Ontario Canada; ^5^ Sandra E Black Centre for Brain Resilience and Recovery, Hurvitz Brain Sciences Program Sunnybrook Research Institute Toronto Ontario Canada; ^6^ Department of Medical Biophysics University of Toronto Toronto Ontario Canada; ^7^ Computational Radiology & Artificial Intelligence Unit, Department of Radiology and Nuclear Medicine Oslo University Hospital Oslo Norway

**Keywords:** bipolar disorder, cardiovascular disease, neurostructure, polygenic risk, youth

## Abstract

**Introduction:**

Bipolar disorder (BD), characterized by anomalous neurostructural phenotypes, is also strongly associated with cardiovascular disease. Here we examined polygenic risk for coronary artery disease (CAD) in relation to gray matter structure in youth BD.

**Methods:**

Youth participants (mean age 17.1 years; *n* = 66 BD, *n* = 45 healthy controls [HC]) underwent T1‐weighted magnetic resonance imaging. CAD polygenic risk scores (CAD‐PRS) were calculated using independent, adult genome‐wide summary statistics. Covariate‐adjusted vertex‐wise analyses examined the association of CAD‐PRS with cortical volume, thickness, and surface area (SA) in the overall sample, and within BD and HC groups. Additional region‐of‐interest (ROI) analyses were conducted to examine the anterior cingulate cortex (ACC), amygdala, and hippocampus. Exploratory sex‐stratified analyses were also undertaken.

**Results:**

In the overall sample, higher CAD‐PRS was associated with lower right inferior temporal gyrus volume (*β* = −0.32, *p* = 0.03). There were also negative associations between CAD‐PRS and brain structure within BD (5 cortical thickness clusters) and HC (1 SA cluster). Within the BD group, sex‐stratified analyses revealed significant findings for females, but not for males. ROI analyses revealed a nominal association of higher CAD‐PRS with lower ACC thickness in the BD group (*β* = −0.31, *p*_uncorrected_ = 0.05, *p*_corrected_ = 0.20).

**Conclusion:**

Higher CAD‐PRS was associated with lower regional gray matter structure in youth, in regions implicated in BD. Findings were more pronounced in the BD group, particularly among females, and related to cortical thickness specifically. Future longitudinal studies are needed to examine the association of CAD‐PRS with neurodevelopmental changes over time and to discern mechanisms underlying the observed findings.

## 1 Introduction

Bipolar disorder (BD) is a leading cause of functional disability worldwide [1]. BD commonly onsets in adolescence, and in such early‐onset cases, there are increased rates of psychiatric comorbidity and symptom burden [2]. Additionally, there is widely replicated international evidence of increased risk and premature onset of cardiovascular disease (CVD) among individuals with BD [3, 4]. Onset of CVD occurs up to 17 years prematurely in BD [5], and standardized mortality ratios for CVD are elevated in these individuals relative to a healthy population [3]. This is particularly evident in women with BD, who demonstrate higher standardized mortality ratios for CVD as compared to males with BD [6]. Traditional cardiovascular risk factors (e.g., blood pressure, lipids) are common in BD in treated and untreated samples, and several mood‐stabilizing medications increase this risk. Adverse lifestyle behaviors (e.g., smoking, sedentary lifestyle, binge‐eating) are also common in BD. Nonetheless, the elevated cardiovascular risk in BD is in excess of what can be explained by these factors [4, 5]. Coronary artery disease (CAD) is the most common type of heart disease and the leading cause of death worldwide. Adults with BD have a greater prevalence of CAD [7] and at least double the mortality due to CAD relative to the general population [4].

While anomalous neurostructural metrics in youth and adults with BD are well established, there is a growing body of literature demonstrating that CAD is also associated with neurostructural phenotypes. Specifically, adults with CAD exhibit significantly smaller total brain volume and lower regional gray matter metrics [8, 9, 10]. Since these studies have been undertaken after CAD is clinically evident, it is not clear whether the neuroimaging correlates of CAD are due to downstream circulatory effects of atherosclerosis or due to shared genetic liability.

The discovery of common genetic variants associated with CAD has enabled the development of polygenic risk scores (PRS) [11]. In adults, CAD‐PRS significantly predicts CAD over and above traditional cardiovascular risk factors [12]. There is preliminary evidence of a genetic overlap between CAD and BD, with a recent study based on genome‐wide association data for BD (*n* = 51,710) and CAD (*n* = 332,477) demonstrating an overlap of 900 genetic variants between BD and CAD and a concordant effect for 70% of the shared variants [13]. Furthermore, there is evidence that adults with BD have elevated CAD‐PRS, even after controlling for the presence of CAD [14]. Emerging studies have also started to uncover links between cardiovascular PRS and brain‐related phenotypes. Specifically, one adult study demonstrated that CAD‐PRS is associated with greater whole‐brain volume atrophy in those with mild cognitive impairment and greater prevalence and volume of white matter hyperintensities in the occipital lobe in those with Alzheimer's disease [15].

Most recently, a seminal publication in > 40,000 participants from the UK Biobank and Biobank Japan demonstrated that cardiac MRI metrics were significantly associated with gray matter structure, white matter integrity, and functional connectivity [16]. Furthermore, the study reported significant genetic correlations between adverse cardiac MRI features and neuropsychiatric disorders, including BD, which were determined to be genetically causal [16]. Many of these cardiac MRI traits were genetically associated with CAD, suggesting that the comorbidity of CAD and BD is based on a shared genetic etiology that maps onto the brain.

Despite emerging studies on the link between cardiovascular genetics and psychiatric disorders, there remains a paucity of studies focusing on youth. Focusing on cardiovascular genetics in youth BD provides a unique opportunity to understand the genetic underpinnings of CVD and BD in a sample that is undergoing major developmental changes in neural networks while minimizing the effects of aging and related diseases. As such, this study assessed CAD‐PRS in relation to neurostructural metrics in youth with and without BD. Given the importance of sex differences in the epidemiology, treatment, and outcomes of CVD [17], within‐sex analyses were also undertaken for females and males.

## 2 Materials and Methods

### 2.1 Participants

This study included 111 youth between the ages of 13–21 years and were genetically European. BD participants (type I, II, or other specified bipolar disorder [OSBD]) were recruited through a subspecialty clinic at an academic health science center in Toronto, Ontario, Canada. HC were recruited from the community through advertisements. Exclusion criteria included: known cardiac conditions; autoimmune or inflammatory conditions; taking anti‐inflammatory, anti‐platelet, anti‐lipidemic, anti‐hypertensive, or hypoglycemic agents; infectious illness in the 14 days before study visit; any MRI contraindications; any severe neurological or cognitive impairment, or inability to provide informed consent. Additionally, HC participants had no major or recent psychiatric disorders (no lifetime mood or psychotic disorders, no recent alcohol or drug dependence in the past 3 months, and no recent anxiety disorders within the past 3 months) and no first‐ or second‐degree family history of BD or psychotic disorders.

Written informed consent was obtained from all participants, as well as their parent(s) or guardian(s). Ethical approval was granted by Sunnybrook Research Institute Research Ethics Board and subsequently by the Centre for Addiction and Mental Health (CAMH) Research Ethics Board following the Centre for Youth Bipolar Disorder's relocation to CAMH. Detailed methods regarding study data collection can be found in Supporting Information.

### 2.2 Diagnostic Interview and Symptom Ratings

Diagnostic and clinical information was obtained via interview with youth and parent(s) using the Kiddie‐Schedule for Affective Disorders and Schizophrenia for School‐Age Children, Present and Lifetime version (K‐SADS‐PL) [18]. The K‐SADS‐PL is a semi‐structured interview with both parent and youth that is used to determine present and lifetime history of psychiatric illness in children and adolescents between the ages of 7 and 18 years, according to the Diagnostic and Statistical Manual of Mental Disorders, Fourth Edition (DSM‐IV) (APA, 2000) criteria. Diagnoses in this study were based on the DSM‐IV criteria, as participants were enrolled from 2014 to 2019, and the DSM‐5 version of the K‐SADS‐PL was not available until 2016. Diagnosis of OSBD was based on operationalized criteria from the Course and Outcome of Bipolar Illness in Youth (COBY) study for duration of symptoms (minimum 4 h/day) and number of hypomanic days (minimum 4 in lifetime), while retaining DSM‐5 symptom count requirements (i.e., 3 symptoms when elation was the primary symptom, 4 symptoms when irritability was the primary symptom) [19]. Lifetime use of lithium and SGA was ascertained via the K‐SADS‐PL and was computed as a “yes” or “no” variable.

All interviews were performed by trained study personnel with either a Bachelor's or Master's degree in a health‐related field and who completed comprehensive K‐SADS‐PL training under the supervision of the senior author (B.I.G.), a licensed child and adolescent psychiatrist. All diagnostic and symptom ratings were reviewed and confirmed by a licensed child and adolescent psychiatrist.

### 2.3 Clinical Methods

Comorbid diagnoses and clinical characteristics (e.g., psychosis, psychotropic, and psychosocial treatment history) were collected during the K‐SADS interview. Mood symptoms were assessed through the K‐SADS Mania Rating Scale and the K‐SADS Depression Rating Scale [20, 21]. Age of BD onset was defined as the age at which the individual first experienced an episode of mania or hypomania according to DSM‐IV criteria, or when study criteria for OSBD were met. “Any Anxiety Disorders” included generalized anxiety disorder, separation anxiety disorder, agoraphobia, and anxiety disorder not otherwise specified. SUD included alcohol or drug abuse or dependence. Lifetime nicotine use was ascertained via the K‐SADS‐PL and was computed as a “yes” or “no” variable. K‐SADS‐PL post‐traumatic stress disorder screening questions were used to obtain information regarding lifetime history of sexual and/or physical abuse. Socioeconomic status was calculated using the Hollingshead Four‐Factor Index [22]. The family psychiatric history of all first‐ and second‐degree relatives was determined using the Family History Screen. Participants' global functioning over the current period (past month), most severe past, and highest level in the past year were measured by the Children's Global Assessment Scale (CGAS) [23]. Pubertal status was assessed based on Tanner Staging (1–5; 1 = no sexual maturation, 5 = full sexual maturation) [24].

### 2.4 Anthropometric Data Collection

Participant anthropometric measures were collected at intake. Body weight was measured in kilograms (kg) using a Conair digital scale. Weight measurements were deducted by 1.3 kg for participants wearing long pants and long‐sleeved shirts, 1.1 kg for short pants or short‐sleeved shirts, or 0.9 kg for both short pants and short‐sleeves. Height was measured in centimeters (cm) using a wall‐mounted stadiometer. Waist circumference and body mass index (BMI) were calculated using standard guidelines [25]. Blood pressure was taken using a Life Source Digital Blood Pressure Monitor after the participant had rested for 10 min. All measurements were repeated twice to ensure accuracy; if there was a difference of > 1 cm (height) or > 0.3 kg (weight) between the two measurements, a third measurement was taken.

### 2.5 Polygenic Risk Scores

A saliva sample (~2 mL) was collected from each participant in an Oragene OG‐500 DNA kit (DNA Genotek Oragene‐500 kits; DNA Genotek Inc., Ottawa, Canada). Participants were instructed to abstain from eating, drinking, smoking, and chewing gum 30 min prior to saliva collection. Detailed methods regarding DNA extraction, genotyping, genetic data quality control, and whole‐genome imputation can be found in Supporting Information.

CAD‐PRS was derived from the summary statistics of a GWAS meta‐analysis of CAD in 185,000 adults of European ancestry [26]. CAD‐PRS was calculated using PRS‐CS‐auto, a Bayesian‐based method that places a continuous shrinkage prior on the effect sizes of SNPs in the discovery GWAS summary statistics [27]. PRS‐CS‐auto was implemented using the software default settings and with the LD reference panel provided with the PRS‐CS software, which is computed using the 1000 Genomes European samples and HapMap3 SNPs. PLINK 1.9 was used to weigh all SNPs by their effect sizes calculated using PRS‐CS‐auto and sum all SNPs into a PRS for each individual in the target cohort. The derived PRS was subsequently standardized to a mean of 0 and a SD of 1.

### 2.6 Image Acquisition and Processing

T1‐weighted images were collected using a 3 Tesla (3 T) Philips Achieva system with an 8‐channel head receiver coil and body coil transmission (Philips Medical Systems, Best, Netherlands). The acquisition parameters were as follows: repetition time (TR) 9.5 ms, echo time (TE) 2.3 ms, inversion time (TI) 1400 ms, spatial resolution 0.94 × 1.17 × 1.2 mm (nearly 1 mm isotropic), 256 × 164 × 140 matrix, flip angle 8°, scan duration 8 min 56 s.

Image processing of the T1‐weighted images was performed using FreeSurfer (V6.0) software (http://surfer.nmr.mgh.harvard.edu.myaccess.library.utoronto.ca/) [28]. The detailed image processing protocol can be found in Supporting Information.

### 2.7 Statistical Analysis

Demographic and clinical group differences were evaluated using IBM SPSS Version 27. Group differences were evaluated using *t*‐tests for continuous variables, Kruskal–Wallis *H*‐tests for ordinal variables, and chi‐squared tests for categorical variables. Statistical significance was set at two‐sided *p* < 0.05.

General linear models (GLMs) were used to examine the main effects of CAD‐PRS on each gray matter metric (cortical volume, SA, and thickness). Primary analyses were conducted in the combined sample of BD and HC to maximize power, and because we anticipated the direction of any association would be similar in both diagnostic groups. Secondary analyses further examined the associations within the BD group and the HC group separately. Age, sex, and the first two genetic principal components were included in the models as covariates [29]. Sensitivity analysis additionally controlled for all 10 genetic principal components. Intracranial volume was also included as a covariate when examining volume and SA.

The analytic approach included vertex‐wise analyses alongside region‐of‐interest (ROI) analyses. Vertex‐wise analyses were performed using FreeSurfer 6.0 (http://surfer.nmr.mgh.harvard.edu/) [30]. Results were thresholded at *p* < 0.05 and were corrected for multiple comparisons using permutation testing within the FreeSurfer package (10,000 permutations). Cluster‐wise *p*‐values were then calculated as the probability of detecting a cluster of that size by chance and reported for each significant cluster. Corresponding gray matter regions were identified using the Desikan–Killiany atlas. The *β* values for the association of CAD‐PRS with gray matter metrics for the significant clusters were then calculated in SPSS using the aforementioned GLMs. ROI analyses were performed to examine the association between CAD‐PRS and three ROIs associated with BD: anterior cingulate cortex (ACC), amygdala, and hippocampus. Finally, exploratory sex‐stratified analyses of whole‐brain clusters and the three study ROIs were conducted using the aforementioned GLMs.

Vertex‐wise results were corrected for multiple comparisons using a family‐wise error rate within each imaging modality (i.e., within volume, surface area, and thickness, uncorrected across the different modalities). The Benjamini, Krieger, and Yekutieli two‐stage step‐up method of controlling for false discovery rate (FDR) was used to correct for multiple comparisons in the ROI analyses [31].

## 3 Results

### 3.1 Demographic and Clinical Characteristics

Participant demographic and clinical characteristics are presented in Tables 1, S1, and S2. Compared to the HC group, the BD group had a greater proportion of female participants and a higher mean Tanner stage, along with more severe psychiatric clinical characteristics. Additionally, the BD group had significantly higher BMI, lower intracranial volume, lower total gray matter volume, and lower ACC thickness than the HC group.

**TABLE 1:** Demographic and physiological characteristics of participants in the overall sample.

	BD (*n* = 66)	HC (*n* = 45)	Statistic^a^	*p*	Effect size^b^
Demographics					
Age, years	17.3 ± 1.4	16.8 ± 1.8	1.24	0.20	0.25
Female	45 (68.2)	21 (46.7)	5.14	0.02^c^	0.23
Socioeconomic status	4.2 ± 0.9	4.4 ± 1.0	1.29	0.26	0.003
Intact family	39 (60.0)	30 (66.7)	0.51	0.48	0.07
Tanner stage	4.5 ± 0.7	4.2 ± 0.6	4.53	0.03^c^	0.03
IQ	108.7 ± 12.7	111.4 ± 13.5	0.79	0.43	0.21
Physiological characteristics					
Waist circumference (cm)	79.1 ± 10.5	75.6 ± 7.7	1.93	0.06	0.38
Adjusted BMI (kg/m^2^)	23.9 ± 4.7	21.8 ± 2.9	5.43	0.02^c^	0.04
Systolic BP (mmHg)	108.9 ± 11.4	108.1 ± 14.6	0.32	0.75	0.06
Diastolic BP (mmHg)	68.5 ± 7.1	66.3 ± 8.0	1.50	0.14	0.29
Intracranial volume (mm^3^)	1,504,700.5 ± 151,167.0	1,569,717.0 ± 159,928.2	2.17	0.03^c^	0.42
CAD‐PRS	0.03 ± 1.1	0.07 ± 1.0	0.18	0.86	0.04

*Note:* Values for all continuous and ordinal variables are written as mean ± standard deviation; categorical variables are written as *n* (% within group).

Abbreviations: BD = bipolar disorder; BMI = body mass index; BP = blood pressure; CAD‐PRS = coronary artery disease polygenic risk scores; HC = healthy controls; IQ = intelligence quotient.

^a^ Statistic = *t* for dimensional variables, *H* (1 degree of freedom) for ordinal variables, or *χ* ^2^ for categorical variables.

^b^ Effect size = Cohen's *d* for *t* test, partial *η* ^*2*^ for *H* test, or Cramer's *V* for *χ* ^2^ test.

^c^ Significance at *α* = 0.05.

### 3.2 PRS and Gray Matter Structure

**FIGURE 1:**
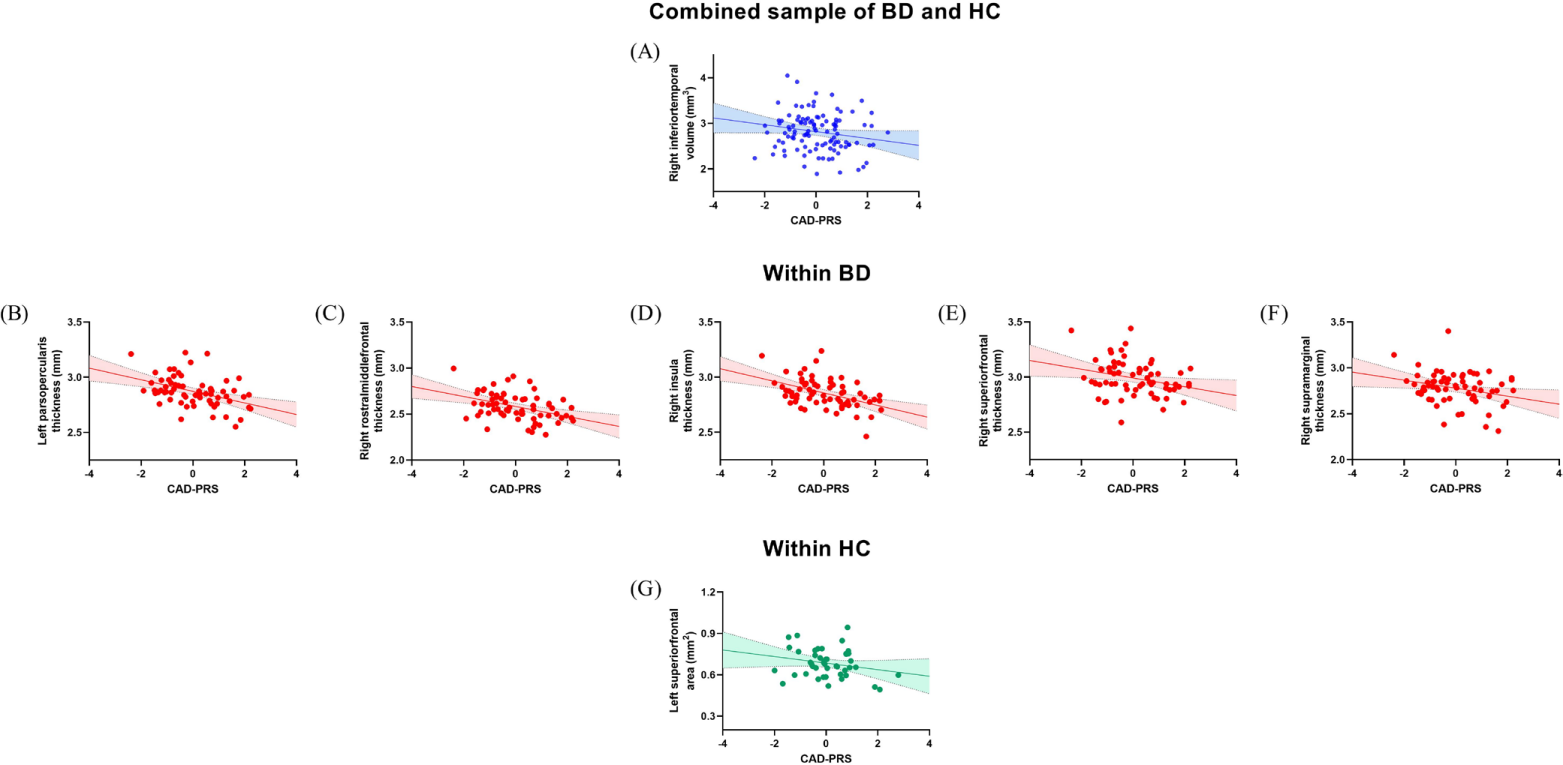
Association between CAD‐PRS and gray matter structure. Within the combined sample of BD and HC, higher CAD‐PRS was significantly associated with smaller cortical volume in the right inferior temporal gyrus (A). Within the BD group, higher CAD‐PRS was significantly associated with smaller cortical thickness in the left pars opercularis (B), right rostral middle frontal gyrus (C), right insula (D), right superior frontal gyrus (E), and right supramarginal gyrus (F). Within the HC group, higher CAD‐PRS was associated with smaller surface area in the left superior frontal gyrus (G).

The main effects of CAD‐PRS on gray matter structure in the combined sample and within the BD and HC subgroups are presented in Table 2 and Figure 1. In the combined sample, higher CAD‐PRS was associated with significantly smaller right inferior temporal gyrus volume (*β* = −0.32, *p* = 0.03). Within the BD group, higher CAD‐PRS was associated with significantly smaller cortical thickness in the left pars opercularis (*β* = −0.60, *p* = 0.003), right rostral middle frontal gyrus (*β* = −0.54, *p* = 0.008), right insular cortex (*β* = −0.65, *p* = 0.009), right superior frontal gyrus (*β* = −0.54, *p* = 0.03), and right supramarginal gyrus (*β* = −0.56, *p* = 0.04). Within HC, higher CAD‐PRS was associated with significantly smaller left superior frontal gyrus SA (*β* = −0.41, *p* = 0.02).

**TABLE 2:** Characteristics of clusters identified in vertex‐wise analyses with significant associations between CAD‐PRS and gray matter structure.

Cortical metrics	MNI coordinates			Cluster size	*β*	*p*	Main region	Additional regions
	*x*	*y*	*z*					
Main effect of CAD‐PRS in the overall sample								
Volume	46.5	−6.6	−40.5	810.65	−0.32	0.03	Right inferior temporal gyrus	Fusiform gyrus, middle temporal gyrus
Main effect of CAD‐PRS within the BD group								
Thickness	−46.3	4.7	15.1	1264.34	−0.60	0.003	Left pars opercularis	Insula, transverse temporal gyrus, superior temporal gyrus, precentral gyrus
	34.1	42.8	23.1	1012.72	−0.54	0.008	Right rostral middle frontal gyrus	Superior frontal gyrus
	37.0	−12.9	20.8	983.18	−0.65	0.009	Right insula	Postcentral gyrus, precentral gyrus, superior temporal gyrus, transverse temporal gyrus
	8.5	10.1	64.9	815.18	−0.54	0.03	Right superior frontal gyrus	—
	60.2	−27.7	34.4	791.29	−0.56	0.04	Right supramarginal gyrus	Postcentral gyrus
Main effect of CAD‐PRS within the HC group								
Surface area	−20.8	22.4	39.5	1547.64	−0.41	0.02	Left superior frontal gyrus	Caudal middle frontal gyrus, rostral middle frontal gyrus

*Note:* Cluster‐wise *p*‐value threshold set at *α* = 0.05.

Abbreviations: BD = bipolar disorder; CAD = coronary artery disease; HC = healthy control; MNI = Montreal Neurological Institute; PRS = polygenic risk score.

Results remained significant after controlling for all 10 genetic principal components in a sensitivity analysis.

### 3.3 ROI Analyses

**FIGURE 2:**
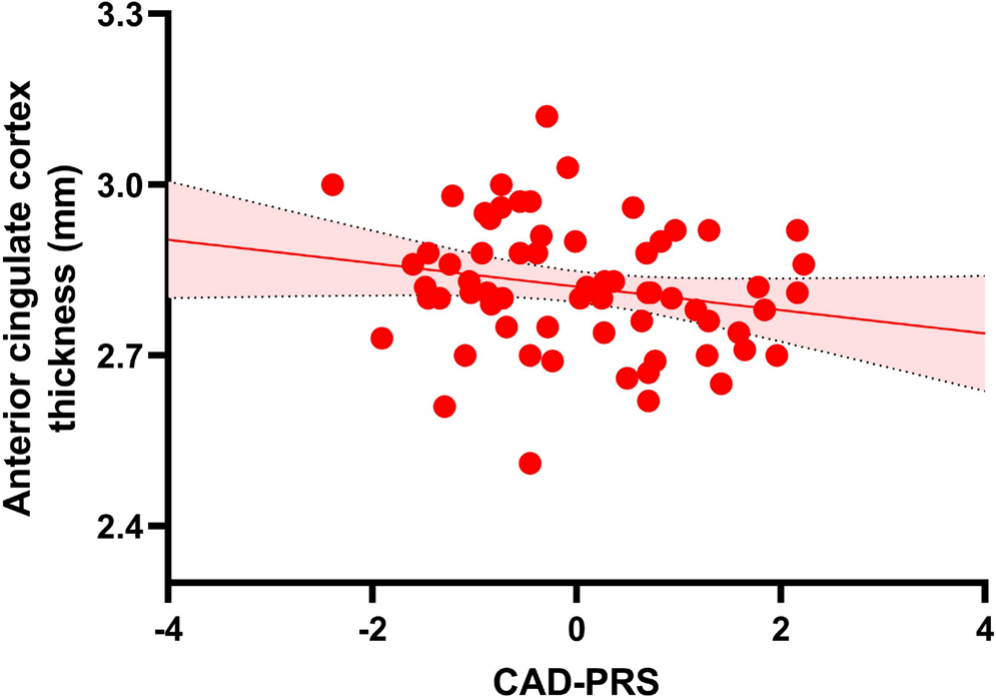
Association between CAD‐PRS and the ACC. In the BD group, higher CAD‐PRS was significantly associated with lower cortical thickness in the ACC.

ROI analyses examining the effects of CAD‐PRS on structural phenotypes of the ACC, amygdala, and hippocampus are presented in Table S3 and Figure 2. Within the BD group, higher CAD‐PRS was nominally associated with smaller cortical thickness in the ACC (*β* = −0.31, *p*_uncorrected_ = 0.053). Findings did not remain significant after correcting for multiple comparisons (*p*_corrected_ = 0.26). No other associations were significant.

### 3.4 Sex‐Stratified Analyses

Table 3 presents exploratory sex‐stratified analyses for the vertex‐wise results. The results observed in the primary analyses within the BD group were significant in females but not in males. The difference between sexes was particularly robust in the right superior frontal gyrus (females: *β* = −0.61, *p* < 0.001; males: *β* = −0.39, *p* = 0.23) and the right supramarginal gyrus (females: *β* = −0.72, *p* < 0.001; males: *β* = −0.05, *p* = 0.89). The results observed in the primary analyses within the combined sample and within HC remained significant in, but did not differ meaningfully between, females and males.

**TABLE 3:** Association between CAD‐PRS and gray matter structure stratified by sex.

Cortical metrics	Females		Males		Main region	Additional region(s)
	*β*	*p*	*β*	*p*		
Main effect of CAD‐PRS in the overall sample						
Volume	−0.31	0.02^a^	−0.43	0.02^a^	Right inferior temporal gyrus	Fusiform gyrus, middle temporal gyrus
Main effect of CAD‐PRS within the BD group						
Thickness	−0.60	< 0.001^a^	−0.54	0.09	Left pars opercularis	Insula, transverse temporal gyrus, superior temporal gyrus, precentral gyrus
	−0.60	< 0.001^a^	−0.45	0.17	Right rostral middle frontal gyrus	Superior frontal gyrus
	−0.68	< 0.001^a^	−0.54	0.09	Right insula	Postcentral gyrus, precentral gyrus, superior temporal gyrus, transverse temporal gyrus
	−0.61	< 0.001^a^	−0.39	0.23	Right superior frontal gyrus	—
	−0.72	< 0.001^a^	−0.05	0.89	Right supramarginal gyrus	Postcentral gyrus
Main effect of CAD‐PRS within the HC group						
Surface area	−0.52	0.01^a^	−0.53	0.005^a^	Left superior frontal gyrus	Caudal middle frontal gyrus, rostral middle frontal gyrus

Abbreviations: BD = bipolar disorder; CAD = coronary artery disease; HC = healthy control; PRS = polygenic risk score.

^a^ Significance at *α* = 0.05.

There were no significant sex differences in the ROI results.

## 4 Discussion

This study examined the association between CAD polygenic risk and gray matter structure in youth with BD. All significant findings reflected a consistent pattern whereby higher CAD‐PRS was associated with lower regional neurostructural metrics. Findings were especially prominent in youth with BD, particularly in females. Importantly, these findings are evident in a young sample, early in their course of BD and decades before typical onset of CAD. These findings have implications for the conceptualization of the link between BD and CAD.

In vertex‐wise analyses, higher CAD‐PRS was associated with smaller cortical volume of the right inferior temporal gyrus. This region is involved in high‐order visual processing and emotion regulation, neurocognitive domains that are commonly impaired in BD [32]. There is evidence of reduced inferior temporal lobe volume in adults with CAD [8] and heart failure [33] as compared to controls, and in association with cardiovascular risk factors (i.e., type II diabetes, hypertension, obesity) [34]. Similarly, reduced inferior temporal gyrus volume and SA have been reported in youth and young adults with BD as compared to controls [35, 36].

Our primary findings in the BD group demonstrated that higher CAD‐PRS was associated with smaller cortical thickness in the left pars opercularis, right rostral middle frontal gyrus, right insula, right superior frontal gyrus, and right supramarginal gyrus. Additional clusters included the temporal gyrus, precentral gyrus, and postcentral gyrus. Uncorrected ACC ROI analysis demonstrated a similar negative trend (*p* = 0.053) between CAD‐PRS and ACC thickness in the BD group. Together, these regions are relevant to psychopathology and have been implicated in BD specifically, among both adults and youth [36, 37]. While there is far less neuroimaging research related to CAD as compared to BD, there is nonetheless a small literature among adults with CAD showing reduced gray matter structure in overlapping regions [8, 9, 10]. The relevance of the observed structural alterations to neurocognition is well‐documented. Reduced cortical thickness in the superior frontal gyrus, insula, and supramarginal gyrus has been associated with impairments in executive function, attention, and working memory [38, 39]—domains affected in both BD and CVD.

Our group has previously demonstrated in partially overlapping youth samples that traditional cardiovascular risk factors (e.g., lipids, blood pressure) and novel vascular measures (e.g., reactive hyperemia index, retinal vascular caliber) are relevant to brain structure [40, 41, 42, 43]. Unlike the prior studies that had more variability within the healthy youth, findings in the current study had similar directions in both BD and HC. Furthermore, the findings were more prominent in BD (five significant clusters) versus HC (one significant cluster). The findings in this study, while preliminary and warranting replication, build upon the prior youth literature by suggesting that genetic drivers of CVD may also be driving brain dysfunction associated with BD [9].

The question arises as to which biological pathways may be mediating the observed effects. Risk loci for CAD include those involved in inflammation, lipid metabolism, blood pressure, and vascular remodeling [44]. CAD‐related processes have also been implicated in BD, including inflammation, oxidative stress, cardiometabolic dysfunction, mitochondrial dysfunction, and microvascular dysfunction [2, 3]. Given that this combination of processes is related to both CAD and to BD, one can speculate that these processes are also relevant to mediators of the association of CAD‐PRS with brain structure in the current study. Together, these processes could affect gray matter structure via a combination of reduced cerebral blood flow and chronic relative hypoperfusion, neuronal dysfunction, impaired neurovascular coupling, reduced synaptic plasticity and cellular efficiency, and accelerated neurodegeneration [45]. This study forms the basis for future mechanistic studies, including future analyses evaluating overlapping genetic variants that may be most relevant to the vascular‐bipolar connection.

Our analyses in the HC group demonstrated that higher CAD‐PRS was associated with lower left superior frontal gyrus area, a region that is implicated in working memory and spatial processing [46]. There is some literature demonstrating gray matter loss and reduced brain activity in this region in older adults with CVD (i.e., coronary heart disease, ischemic heart disease, heart failure) [47, 48] and decreased cerebrovascular reactivity in older CAD patients specifically [49]. Our findings in a healthy youth sample demonstrate that genes implicated in CAD have an influence on brain structure that precedes the clinical onset of CAD.

It is worth noting that while the direction for results was consistent across all analyses, including the combined sample, within BD, and within HC (i.e., negative correlation between CAD‐PRS and imaging phenotype), the imaging phenotype differed by diagnosis. Specifically, cortical volume within the right inferior temporal gyrus was implicated in the combined sample, whereas this finding was not replicated within the BD or HC subgroup vertex‐wise analyses. Given that the effect sizes within the right inferior temporal gyrus were similar across the combined sample (β = −0.32), within‐BD (β = −0.33), and within‐HC (β = −0.33), we believe the lack of statistical significance in the subgroup analyses is attributable to lower statistical power.

The fact that BD group findings related to cortical thickness aligns with consistent evidence of reduced cortical thickness in the superior temporal gyrus, superior frontal gyrus, insula, and the ACC in BD [50]. Similarly, a recent study in adults with CAD demonstrated reduced cortical thickness in the insula, anterior prefrontal cortex, superior temporal gyrus, posterior middle frontal gyrus, precentral gyrus, and inferior frontal gyrus, among other regions [10]. Taken together, there is prior evidence that cortical thickness is reduced in both BD and CAD, respectively, and the current study extends this evidence by providing preliminary evidence that higher that CAD‐PRS was associated with reduced cortical thickness in BD.

Finally, the exploratory sex‐stratified analyses in the BD group found that all five clusters remained significant in females, whereas none remained significant in males. The effect sizes were similar for females and males within two of the five clusters; for the remaining clusters, effect sizes were large in females but small to medium in males. These differences may relate in part to the smaller sample size for males. Future studies are warranted to further evaluate sex differences in the association between CAD‐PRS and brain structure in BD. These tentative sex differences are relevant in light of well‐established sex differences in the clinical epidemiology of CVD [17], and in light of the fact that the association of BD with CVD is particularly strong in females [6].

There are some study limitations that should be considered. First, the cross‐sectional design precludes conclusions regarding the timing of the observed associations. In particular, we cannot determine whether BD‐related differences preceded or followed the onset of BD. Second, while the sample size was relatively large for a single‐site study focusing on youth BD, it did not provide adequate power to evaluate small effect sizes, especially for within‐group analyses. This may explain the variability in imaging phenotypes in relation to diagnosis, despite all imaging phenotypes being implicated in all groups. Third, the smaller sample of males as compared to females within the BD group may have led to underpowered sex‐stratified analyses. Fourth, there are sources of residual confounding that were not accounted for in the primary analyses. Finally, since CAD‐PRS scores were based on summary statistics derived from individuals of European ancestry, only participants of European ancestry were considered for the current study.

Despite these limitations, this study demonstrates that polygenic risk for CAD is associated with reduced regional gray matter structure in youth, particularly in youth with BD, in regions implicated in BD. These findings advance understanding of the interface between cardiovascular genetic risk and brain structure in youth BD. The preliminary sex‐related findings provide an impetus for future larger studies. Given that the temporal onset of BD precedes CVD onset by decades, CVD is generally viewed as a downstream effect of BD, driven by mood symptoms (and related biological perturbations), psychotropic medications, and adverse lifestyle. The notion that polygenic risk for CVD may impact the brain, and in turn BD, is almost entirely absent from contemporary research and scientific discourse. This is especially true for youth, an age group that arguably stands to benefit most from preventive and therapeutic implications of heart‐brain associations in BD. Our findings provide an alternate view by demonstrating that increased polygenic risk for CVD is related to brain structure in youth with BD, even before the clinical manifestation of CVD.

Additional studies are needed to build on current findings. Future prospective, repeated‐measures studies would help elucidate when neurostructural changes in association with CAD‐PRS emerge and how these changes progress over time. Larger samples are warranted to enable more comprehensive covariate modeling so that additional factors such as psychiatric comorbidity, psychotropic medications, lifestyle factors, and epigenetics can be included in parallel. Finally, summary statistics from GWAS derived from ancestrally diverse populations are required to better assess the association between CAD‐PRS and brain structure in a sample representative of the general population.

Continued research on this topic has the potential to change the conceptualization of heart‐brain associations among youth with BD by demonstrating that cardiovascular‐brain associations are in part genetically driven. This may ultimately inform clinical practice by demonstrating the value of optimizing cardiovascular health for neuroprotective health, even among individuals without clinical manifestations of CV, and reduce stigma associated with psychiatric illness.

## Author Contributions

N.P.K. primarily wrote the manuscript and performed statistical analyses. C.C.Z. conducted GWAS quality control and PRS calculations. K.G.K. conducted neuroimaging analyses and quality control. M.M. reviewed clinical data. B.I.G. contributed to study conception, design, and assisted with manuscript preparation. M.M. reviewed clinical data and B.J.M. reviewed imaging data. L.T.Y. contributed to study conceptualization and manuscript review. All authors contributed to revisions of the manuscript and have approved the final manuscript.

## Ethics Statement

The authors assert that all procedures contributing to this work comply with the ethical standards of the relevant national and institutional committees on human experimentation and with the Helsinki Declaration of 1975, as revised in 2008. Consent was obtained from all participants and their parent and/or guardian prior to participating. Ethical approval was granted by Sunnybrook Research Institute Research Ethics Board (REB nos. 408‐2011 and 409‐2013). All data was collected at Sunnybrook Research Institute. However, all data was transferred with the Centre for Youth Bipolar Disorder's relocation to the Centre for Addiction and Mental Health (CAMH). Thus, ethics approval was also granted by CAMH Research Ethics Board (REB no. 168/2020 and 165/2020).

## Conflicts of Interest

Dr. Clement C. Zai receives an honorarium for a Medscape review on bipolar disorder genetics. Dr. Benjamin I. Goldstein acknowledges his position as RBC Investments Chair in Children's Mental Health and Developmental Psychopathology at CAMH, a joint Hospital‐University Chair between the University of Toronto, CAMH, and the CAMH Foundation. All other authors declare no conflicts of interest.

## Supporting information


**Data S1:** bdi70065‐sup‐0001‐DataS1.docx.

## Data Availability

The datasets used and/or analyzed during the current study are available from the corresponding author on reasonable request. The data are not publicly available due to privacy or ethical restrictions.
