# Supplementary material for: Neurostructural Correlates of Polygenic Risk for Coronary Artery Disease in Relation to Youth Bipolar Disorder
Source: Bipolar Disord. 2025 Sep 24;27(8):557–66. doi: 10.1111/bdi.70065 (PMC12706564; doi:10.1111/bdi.70065)
Supplement: Supplementary file 1 — Data S1: bdi70065‐sup‐0001‐DataS1.docx. [file BDI-27-557-s001.docx]

**Supporting Information**

*Collection of study data*

Study data were collected and managed using REDCap electronic data capture tools hosted at Sunnybrook Health Sciences Centre and later at the Centre for Addiction and Mental Health. REDCap (Research Electronic Data Capture) is a secure, web-based software platform designed to support data capture for research studies, providing 1) an intuitive interface for validated data capture; 2) audit trails for tracking data manipulation and export procedures; 3) automated export procedures for seamless data downloads to common statistical packages; and 4) procedures for data integration and interoperability with external sources.^1,2^

*DNA extraction and genotyping*

DNA extraction was performed using a chemagic MSM I DNA extractor (Perkin-Elmer, Waltham, MA) as per manufacturer’s instructions. The extracted DNA was quantified using Qubit 2.0 Fluorometer (LifeTechnologies, Toronto, ON, Canada) and diluted to 50 ng/µL for use in downstream genotyping applications. Prior to analysis, all DNA dilutions were assayed using a custom TaqMan genotyping assay (LifeTechnologies, Toronto, ON, Canada) according to manufacturer’s directions for the amelogenin region. This assay was used as a quality control measure to identify any gross errors when manually preparing the dilutions and/or plating the samples (primer and probe sequences available upon request). DNA samples were genotyped on Infinium Global Screening Arrays (v3; Illumina) using iScan Array Scanner following manufacturer’s procedures (Illumina, Vancouver, BC, Canada). All genetic sample processing (DNA extraction and genotyping) was performed by the CAMH Biobank and Molecular Core Facility. Technicians were blinded to study groups.

*Genetic quality control and imputation*

Quality control was performed on genome-wide data. Individuals participants were excluded based on the following criteria: (1) having the same genotype as another participant in the study, (2) having a first- or second- degree relative in the sample, (3) having self-reported sex that differed from the estimated sex derived from the Global Screening Array genotypes, (4) having abnormally high genome-wide heterozygosity (> 3 SD ± sample mean), (5) having more than 5% missing genotype data, (6) having genetically non-European ancestry, or (7) being outliers on the top 10 ancestry informative principal components (> 6 SD ± sample mean). SNPs were excluded based on the following criteria: (1) deviation from the Hardy-Weinberg equilibrium (*p* < 1 x 10^-6^), (2) minor allele frequency < 1%, and (3) missingness rate > 5%. Data were imputed using Minimac4 via the Michigan Imputation Server at CAMH with 1000 Genomes Phase 3 European subgroup (CEU) as reference data. Imputed SNPs were excluded based on the following criteria: (1) minor allele frequencies < 5%, (2) missing genotype rates > 1%, (3) deviation from the Hardy-Weinberg equilibrium (p<10^-6^), or (4) poor imputation quality (imputation information score < 0.7).

*Image processing protocol of the T1-weighted brain images*

T1-weighted images first underwent removal of non-brain tissue via automated skull stripping,^3^ Talairach transformations, parcellation of white and gray matter,^4^ intensity normalization,^5^ tessellation of the gray matter white matter boundary,^6^ and topology correction.^7^ The brain was then inflated to enable registration to a spherical atlas which is based on individual cortical folding patterns to match cortical geometry across subjects.^4^ The registered brain was then mapped to the Desikan-Killiany probabilistic atlas for cortical parcellation.^8^ To facilitate the vertex-wise analysis, surface-based smoothing with a full-width at half-maximum of 15 mm was employed before mapping volumetric, surface area (SA), and thickness data to the canonical template. Three independent raters performed assessments to ensure the quality of images (e.g. contrast between white and grey matter, image quality, and artifacts) and the accuracy of parcellation (e.g. correctly labelled structures).

**References**

1. Harris PA, Taylor R, Thielke R, Payne J, Gonzalez N, Conde JG. Research Electronic Data Capture (REDCap) - A metadata-driven methodology and workflow process for providing translational research informatics support. *J Biomed Inform*. 2009;42(2):377. doi:10.1016/J.JBI.2008.08.010

2. Harris PA, Taylor R, Minor BL, et al. The REDCap Consortium: Building an International Community of Software Platform Partners. *J Biomed Inform*. 2019;95:103208. doi:10.1016/J.JBI.2019.103208

3. Ségonne F, Dale AM, Busa E, et al. A hybrid approach to the skull stripping problem in MRI. *Neuroimage*. 2004;22(3):1060-1075. doi:10.1016/j.neuroimage.2004.03.032

4. Fischl B, Salat DH, Van Der Kouwe AJW, et al. Sequence-independent segmentation of magnetic resonance images. *Neuroimage*. 2004;23 Suppl 1:S69-84. doi:10.1016/J.NEUROIMAGE.2004.07.016

5. Sled JG, Zijdenbos AP, Evans AC. A nonparametric method for automatic correction of intensity nonuniformity in MRI data. *IEEE Trans Med Imaging*. 1998;17(1):87-97. doi:10.1109/42.668698

6. Fischl B, Liu A, Dale AM. Automated manifold surgery: constructing geometrically accurate and topologically correct models of the human cerebral cortex. *IEEE Trans Med Imaging*. 2001;20(1):70-80. doi:10.1109/42.906426

7. Ségonne F, Pacheco J, Fischl B. Geometrically accurate topology-correction of cortical surfaces using nonseparating loops. *IEEE Trans Med Imaging*. 2007;26(4):518-529. doi:10.1109/TMI.2006.887364

8. Desikan RS, Ségonne F, Fischl B, et al. An automated labeling system for subdividing the human cerebral cortex on MRI scans into gyral based regions of interest. *Neuroimage*. 2006;31(3):968-980. doi:10.1016/J.NEUROIMAGE.2006.01.021

**Supplementary Table 1.** Clinical characteristics of participants with bipolar disorder (n=66).

| *Clinical characteristics* | |
| --- | --- |
| BD-I | 21 (31.8)^a^ |
| BD-II | 21 (31.8) |
| BD-NOS | 24 (36.4) |
| Age of BD onset, years | 14.5±2.7 |
| Lifetime psychosis | 16 (24.2) |
| Lifetime suicide attempts | 11 (16.7) |
| Lifetime suicidal ideation | 50 (75.8) |
| Lifetime self-injurious behaviour | 40 (60.6) |
| Lifetime physical abuse | 2 (3.0) |
| Lifetime sexual abuse | 3 (4.5) |
| Lifetime psychiatric hospitalization | 29 (43.9) |
| Depression score – Most severe past episode | 32.6±10.5^b^ |
| Depression score – Current | 16.5±11.3 |
| Mania score – Most severe past episode | 30.6±11.2 |
| Mania score – Current | 10.9±10.3 |
| CGAS – Most severe past episode | 44.0±8.4 |
| CGAS – Highest past year | 67.3±10.6 |
| CGAS – Past month | 65.2±11.0 |
| *Lifetime comorbid diagnosis* | |
| ADHD | 30 (45.5) |
| Any anxiety | 56 (84.8) |
| Oppositional defiant disorder | 20 (30.3) |
| Conduct disorder | 3 (4.5) |
| Substance use disorder | 12 (18.2) |
| Nicotine use | 32 (48.5) |
| Alcohol abuse | 5 (7.6) |
| Alcohol dependence | 4 (6.1) |
| *Family psychiatric history* |  |
| Mania/hypomania | 40 (62.5) |
| Depression | 52 (81.3) |
| Psychosis | 16 (25.0) |
| ADHD | 23 (35.9) |
| Anxiety | 47 (73.4) |
| *Lifetime psychotropic medications* | |
| Any medications | 54 (81.8) |
| SGA | 45 (68.2) |
| Lithium | 14 (21.2) |
| SSRI | 25 (37.9) |
| Non-SSRI | 13 (19.7) |
| Stimulants | 14 (21.2) |
| Valproate | 4 (6.1) |
| Lamotrigine | 16 (24.2) |
| *Current psychotropic medications* | |
| Any medications | 55 (83.3) |
| SGA | 40 (60.6) |
| Lithium | 12 (18.2) |
| SSRI | 7 (10.6) |
| Non-SSRI | 5 (7.6) |
| Stimulants | 4 (6.1) |
| Valproate | - |
| Lamotrigine | 16 (24.2) |

**Note:** ^a^Values for categorical variables are written as n (% within group).

^b^Values for continuous and ordinal variables are written as mean ± standard deviation.

BD=bipolar disorder; NOS=not otherwise specified; CGAS=Children’s Global Assessment Scale; ADHD=attention deficit hyperactivity disorder; SGA=second-generation antipsychotic; SSRI=selective serotonin reuptake inhibitor.

**Supplementary Table 2.** Relevant neurostructural characteristics of participants.

|  | BD  (n=66) | HC  (n=45) | Statistic^†^ | *p* | Effect size^‡^ |
| --- | --- | --- | --- | --- | --- |
| Total GMV (mm^3^) | 712389.5±61936.8 | 749722.2±67393.1 | 3.01 | 0.003* | 0.58 |
| Subcortical GMV (mm^3^) | 61663.8±4515.1 | 63231.3±4765.2 | 1.76 | 0.08 | 0.34 |
| ACC volume (mm^3^) | 19916.5±2662.5 | 20793.0±2424.6 | 1.77 | 0.08 | 0.34 |
| ACC thickness (mm) | 2.8±0.1 | 2.9±0.1 | 2.89 | 0.005* | 0.56 |
| ACC surface area (mm^2^) | 6684.7±846.8 | 6850.3±818.2 | 1.03 | 0.31 | 0.20 |
| Amygdala volume (mm^3^) | 3501.7±344.2 | 3534.0±351.6 | 0.48 | 0.63 | 0.09 |
| Hippocampal volume (mm^3^) | 8484.9±714.1 | 8681.1±757.6 | 1.39 | 0.17 | 0.27 |

**Note:** Values for all continuous variables are written as mean ± standard deviation.

^†^Statistic=t for dimensional variables. ^‡^Effect Size=Cohen’s *d* for t test. *=significance at α=0.05.

BD=bipolar disorder; HC=healthy controls; GMV=grey matter volume; ACC=anterior cingulate cortex.

**Supplementary Table 3.** Association between CAD-PRS and grey matter structure in brain regions-of-interest.

| **Cortical**  **Metrics** | **Anterior Cingulate Cortex** | | | **Amygdala** | | | **Hippocampus** | | |
| --- | --- | --- | --- | --- | --- | --- | --- | --- | --- |
|  | **β** | ***p*** | ***p****_FDR_* | **β** | ***p*** | ***p****_FDR_* | **β** | ***p*** | ***p****_FDR_* |
| *Main effect of CAD-PRS in the overall sample* | | | | | | | | | |
| Volume | -0.02 | 0.85 | 0.89 | -0.04 | 0.70 | 0.89 | 0.09 | 0.29 | 0.76 |
| Thickness | -0.14 | 0.21 | 0.76 | - | - | - | - | - | - |
| Surface Area | 0.05 | 0.51 | 0.89 | - | - | - | - | - | - |
| *Main effect of CAD-PRS within the BD group* | | | | | | | | | |
| Volume | -0.12 | 0.31 | 0.81 | -0.02 | 0.87 | 0.95 | 0.07 | 0.64 | 0.95 |
| Thickness | -0.31 | 0.053* | 0.26 | - | - | - | - | - | - |
| Surface Area | 0.02 | 0.90 | 0.95 | - | - | - | - | - | - |
| *Main effect of CAD-PRS within the HC group* | | | | | | | | | |
| Volume | 0.08 | 0.47 | 0.99 | -0.05 | 0.74 | 0.99 | 0.03 | 0.78 | 0.99 |
| Thickness | 0.01 | 0.94 | 0.99 | - | - | - | - | - | - |
| Surface Area | 0.08 | 0.43 | 0.99 | - | - | - | - | - | - |

**Note.** CAD=coronary artery disease; PRS=polygenic risk score; MNI=Montreal Neurological Institute; FDR=false discovery rate; BD=bipolar disorder; HC=healthy control. *=significance at α=0.05.
